# Construct Validity and Test–Retest Reliability of the Automated Vehicle User Perception Survey

**DOI:** 10.3389/fpsyg.2021.626791

**Published:** 2021-01-25

**Authors:** Justin Mason, Sherrilene Classen, James Wersal, Virginia Sisiopiku

**Affiliations:** ^1^Department of Occupational Therapy, University of Florida, Gainesville, FL, United States; ^2^Department of Civil, Construction, and Environmental Engineering, University of Alabama at Birmingham, Birmingham, AL, United States

**Keywords:** automated vehicles, user perception survey, exploratory factor analysis, Mokken scale analysis, test–retest reliability, technology acceptance

## Abstract

Fully automated vehicles (AVs) hold promise toward providing numerous societal benefits including reducing road fatalities. However, we are uncertain about how individuals’ perceptions will influence their ability to accept and adopt AVs. The 28-item Automated Vehicle User Perception Survey (AVUPS) is a visual analog scale that was previously constructed, with established face and content validity, to assess individuals’ perceptions of AVs. In this study, we examined construct validity, via exploratory factor analysis and subsequent Mokken scale analyses. Next, internal consistency was assessed via Cronbach’s alpha (α) and 2-week test–retest reliability was assessed via Spearman’s rho (ρ) and intraclass correlation coefficient (ICC). The Mokken scale analyses resulted in a refined 20-item AVUPS and three Mokken subscales assessing specific domains of adults’ perceptions of AVs: (a) *Intention to use*; (b) *perceived barriers*; and (c) *well-being*. The Mokken scale analysis showed that all item-coefficients of homogeneity (H) exceeded 0.3, indicating that the items reflect a single latent variable. The AVUPS indicated a strong Mokken scale (*H*_scale_ = 0.51) with excellent internal consistency (α = 0.95) and test–retest reliability (ρ = 0.76, ICC = 0.95). Similarly, the three Mokken subscales ranged from moderate to strong (range *H*_scale_ = 0.47–0.66) and had excellent internal consistency (range α = 0.84–0.94) and test–retest reliability (range ICC = 0.84–0.93). The AVUPS and three Mokken subscales of AV acceptance were validated in a moderate sample size (*N* = 312) of adults living in the United States. Two-week test–retest reliability was established using a subset of Amazon Mechanical Turk participants (*N* = 84). The AVUPS, or any combination of the three subscales, can be used to validly and reliably assess adults’ perceptions before and after being exposed to AVs. The AVUPS can be used to quantify adults’ acceptance of fully AVs.

## Introduction

Throughout the world, 50 million people are injured each year from traffic crashes (World Health Organization [WHO], 2017). Human factors, such as driving with distraction and/or fatigue, contribute to 93% of all traffic crashes (Fagnant and Kockelman, 2015; Piao et al., 2016). Fully automated vehicles (AVs), where the autonomous driving technology system performs all driving tasks (Levels 4 and 5), as defined by the Society of Automotive Engineers (SAE International, 2018), show great promise towards reducing road fatalities, traffic congestion, and fuel consumption (Payre et al., 2014; NHTSA, 2016; SAE International, 2018). This technology will catalyze changes to infrastructure, policies, and public transit which require substantial financial resources and support from policymakers and taxpayers. However, all these potential societal benefits will not be achieved unless these vehicles are widely accepted and adopted by road users. Thus, as we prepare for the potential introduction of AVs into the market, it is important to understand individuals’ perceptions and attitudes toward the use of AVs, and to validate measurement tools used to obtain such perspectives.

Recent studies suggest that a variety of advantages and disadvantages may arise from the emergence of AVs. The adoption of AVs may improve road safety for all road users and enhance mobility for those who are transportation disadvantaged (e.g., elderly or individuals living with disabilities; Yang and Coughlin, 2014). However, AV adoption may increase traffic congestion, due to enhanced transit availability, increased affordability (i.e., offering traveling opportunities for those currently transportation disadvantaged), and unoccupied vehicles traveling to pick up users (Pettigrew et al., 2018). The public is primarily concerned about AVs relating to privacy, security, insurance, and liability, as well as job losses (Taeihagh et al., 2019). The eventuation of these outcomes is dependent on if users understand system capabilities, accept, and adopt this emerging technology; and if policies guide, protect and facilitate safe and secure use of AVs.

A multitude of automotive manufacturers, technology companies, transportation network companies, and institutions are developing innovative technology to address transportation safety and equity for users across the lifespan and mobility spectrum. These developers must create technologies that are safe and efficient, while also acceptable and adoptable by the intended users. Recent studies suggesthat AVs should be safer than human drivers in order for transportation users to adopt and accept this technology (Waycaster et al., 2018; Shladover and Nowakowski, 2019). Thus, the public will be less likely to embrace AVs if they have the same risk level as human driving. Furthermore, individuals increase their expectations for safety when entrusting their personal well-being (W) and safety to an external mechanism such as an AV (Waycaster et al., 2018). Specifically, Liu et al. (2019) found that AVs should be four to five times safer (i.e., 75–80% reduction in traffic fatalities) than human drivers, if they are to be accepted. Although safety is a critical predictor of acceptance, several other factors (i.e., perceived usefulness, perceived ease of use, and trust) also influence users’ behavioral intentions (Classen et al., 2020).

It is still unclear whether AVs will follow traditional vehicle ownership trends (i.e., private AV), automated shared mobility with pooled ridership, on-demand via transportation network companies (i.e., ride-hailing), or fixed routes via public transit (e.g., automated shuttles). Automated vehicle ownership is difficult to predict due to acceptance, policy, economic concerns, technology advancements, ethical considerations, availability, accessibility, and infrastructure needed, to support the uptake. Assuming that the price for using an AV does not restrict use, the primary determinant of adoption is the users’ perception of acceptance. However, no valid and reliable self-report measure of individuals’ perception of acceptance of fully AVs exists that is relevant or appropriate for a broad population across the lifespan. Upon establishing reliability and validity of the Automated Vehicle User Perception Survey (AVUPS), survey responses may be used to inform vehicle manufacturers, policymakers, and transportation engineers to promote acceptance and adoption of AVs.

**FIGURE 1:**
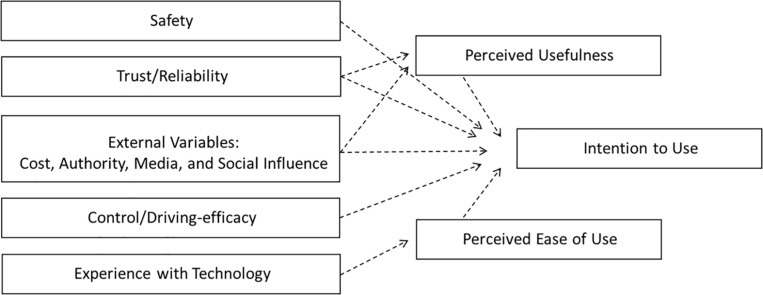
Conceptual model for survey development.

In order to understand adults’ (≥18 years old) perceptions and attitudes toward AV technology adoption, the AVUPS was constructed to quantify such perceptions. Face and content validity for the AVUPS was previously established via focus groups and subject-matter experts (Mason et al., 2020). Survey items were self-generated and adapted from previous models including the *Technology Acceptance Model* (TAM; Davis, 1989), *Unified Theory of Acceptance and Use of Technology* (UTAUT; Venkatesh et al., 2003), *Car Technology Acceptance Model* (CTAM; Osswald et al., 2012), *4P Acceptance Model* (Nordhoff et al., 2016), *Safety Critical Technology Acceptance Model* (SCTAM; Hutchins and Hook, 2017), *Self-driving Car Acceptance Scale* (SCAS; Nees, 2016). The AVUPS was developed to reflect 11 factors (see Figure 1): (a) *Intention to use* (IU); (b) *perceived ease of use*; (c) *perceived usefulness*; (d) *perceived safety*; (e) *trust and reliability*; (f) *experience with technology*; (g) *control and driving-efficacy*; and (h) external variables (i.e., *media, governing authority, social influences*, and *cost*). The arrows represent conceptual pathways that have not yet been tested emmpirically.

Prior to investigating pathways via structural equation modeling, the factor structure must first be established. Factor structure is essential in understanding, scoring, and interpreting the responses on the AVUPS. There are numerous procedures, such as exploratory factor analysis (EFA) or confirmatory factor analysis, which are traditionally used to determine the optimal number of factors to retain in a model. The confirmatory factor analysis is used to verify the factor structure of a set of observed variables and will not be further discussed in this paper due to the overfactoring which is evident in the conceptual model (Figure 1). EFA is suitable approach during early stages of instrument development and can be used to explore the dimensionality of the instrument by identifying relationships between measured variables (Knekta et al., 2019). During EFA, parallel analysis can be employed to compute eigenvalues from the correlation matrix, to determine the number of components to retain for oblimin rotation. The goal of factor rotation is to rotate factors within a multidimensional space to arrive at a solution with best simple structure (i.e., parsimony). Once a plausible solution has been identified by EFA, it is imperative to test this structure utilizing a conceptually different factor analysis method (Wismeijer et al., 2008). Mokken scale analysis (MSA) has shown merit as a complementary tool to EFA and other traditional factor-analytic methods in determining the dimensionality of an item set (Wismeijer et al., 2008; Chen et al., 2016).

Factor structure (i.e., dimensions) from EFA can be used to inform and guide Mokken scaling. This is an alternative approach to conducting exploratory MSA, since the guidelines are not well established for when to test the criteria during the automated item selection procedure (AISP; van der Ark et al., 2008). MSA is a unidimensional procedure that was developed on the basis of the Gutman scaling model and other non-parametric item response theories. MSA assumes that scale items are hierarchically ordered along levels of a latent construct. Compared to traditional factor analysis, MSA is used to investigate dimensionality and evaluate the model at the same time; thus avoiding distortions due to item-score distributions. The Mokken monotone homogeneity model (MHM) assumes unidimensionality, local independence, and latent monotonicity (Mokken, 2011). The unidimensionality assumption of MHM tests the latent structure via an AISP (Sijtsma et al., 2011). In a scale formed by MSA, the sum score of all items can be used as the indicator of the latent trait (Mokken, 2011).

To successfully deploy AVs, adults’ perceptions of acceptance and adoption will need to be validly and reliably quantified to better understand the perceived advantages and disadvantages of this automated technology. Furthermore, this survey may be useful in quantifying changes in users’ perception after being exposed to various forms of fully AVs. The purpose of this study is to report on the construct validity (via factor analysis) and test–retest reliability of the AVUPS, a tool to assess adults’ perceptions of acceptance to fully AVs (SAE Level 4 and 5). Therefore, the study had two objectives: (a) Establish the factor structure of the AVUPS using MSA; and (b) assess internal consistency and 2 weeks test–retest reliability of the AVUPS.

## Materials and Methods

The Institutional Review Board approved this study (IRB201801988) as a subsection of a parent study that investigated older drivers’ perceptions of AVs. All participants provided their written consent or waived consent to participate in the study. The AVUPS was distributed online using Amazon Mechanical Turk (MTurk). Amazon MTurk provided access to a virtual community of workers from different regions of the country with varying backgrounds, who are willing to complete human intelligence tasks (HITs). The researchers of this study submitted a HIT and interested MTurk workers responded using the survey link which directed them to Qualtrics.

### Participants and Sampling

Survey responses from 312 adults living in the United States were used to assess the factor structure and psychometric properties of the AVUPS. The requirements for the MTurk respondents were that they had to be living in the U.S. and have attempted at least 1,000 HITs with a successful completion of at least 95% of their attempted HITs (i.e., Master Workers). The first HIT was completed by 137 participants and they were asked to complete the survey again in 2 weeks. After 2 weeks, 84 participants (61% response rate) completed the survey again. This dataset was used to assess test–retest reliability of the survey. The follow-up responses for the 84 participants were not entered into the factor analysis. A third batch of 65 respondents completed the survey to provide the research team with an adequate sample size for factor analysis (i.e., >250 responses; Straat et al., 2014; Watson et al., 2018). MTurk survey responses from the first and third batch (*n* = 202) were aggregated with survey responses (*n* = 110) collected at baseline from participants eligible (i.e., ≥65 years old; valid driver’s license; no signs of cognitive impairment via the Montreal Cognitive Assessment) and enrolled in an ongoing AV Demonstration study (Classen et al., 2020), resulting in a final sample of 312 participants (*M*_age_ = 47.84, SD_age_ = 18.77; 59% male; 21% non-Caucasian; 2% Hispanic/Latinx).

### Instrumentation

The AVUPS developed by Mason et al. (2020), measures adults’ perceptions of AVs to assess the effects of being exposed to AV technology. Items were ordered thematically in relation to the domains they were intended to represent, to enhance internal consistency reliability (Melnick, 1993). The AVUPS contained 28 visual analoge scale (VAS) items, placed on a 100 mm horizontal line with verbal anchors on the extremes, ranging from disagree to agree. Respondents rated their perceptions by making a mark (i.e., vertical slash) corresponding to their level of agreement/disagreement. The distance between the marked point and the origin of the line is measured to quantify the magnitude of the response. MTurk respondents that completed this process on-line used a sliding scale function in Qualtrics. Additionally, four open-ended items were used to allow individuals to consider and provide their own ideas, thoughts, and feelings (Creswell and Clark, 2011). Prior to responding to items, participants were prompted by the statement:

“An automated vehicle (i.e., self-driving vehicle, driverless car, and self-driving shuttle) is a vehicle that is capable of sensing its environment and navigating without human input. Full-time automation of all driving tasks on any road, under any conditions, and does not require a driver nor a steering wheel.”

### Analysis

Data processing was carried out in RStudio (RStudio, Boston, MA, United States) with R version 4.0.2 (R Core Team, 2020), using the psych and mokken packages. The measurement model was built using a two-stage approach consisting of an EFA and MSA. An EFA was employed to extract the fundamental dimensions of users’ perceptions of AVs and compared those to the conceptual model (Figure 1). The criterion for loading and cross loading was set at 0.4, and based on this, items were removed from the subscales. This iterative and automated process was repeated until a simple structure was achieved where loadings were maximized on putative factors and minimized on the others. Items comprising factors that emerged from the EFA were entered as separate Mokken scales as well as inputting all 28 items into a MSA. Due to negative loading, nine items were reverse scored using the *paste0* function in R. Internal consistency and construct reliability were assessed using Cronbach’s alpha and composite reliability, respectively, both at a factor-level and a scale-level.

The measurement model was built and refined using MSA as it can be used to analyze polytomous items (Sijtsma et al., 2011). MSA was employed to extract the fundamental dimensions of users’ perceptions of AVs via an AISP that partitions a set of data into Mokken scales. MSA evaluates whether a set of items is consistent with the MHM and thus constitutes a scale (Sijtsma and Molenaar, 2002; Straat et al., 2012). The underlying assumptions of MHM are unidimensionality (i.e., single latent trait), local independence (i.e., item responses do not affect other item responses), and latent monotonicity (i.e., probability of a particular response level is a monotonically non-decreasing function of the latent trait; Sijtsma and van der Ark, 2017). The MHM indicates that an item’s score increases as the trait increases, and this is described by the item response curve (IRC). The MHM is important because it justifies ordering respondents according to their raw accumulated scores. The scalability of the scale is measured by Loevinger’s coefficient of homogeneity (*H*). Scalability strength can be judged by the scalability coefficients, such as *H*_i_ (item), measuring precision of item discrimination showing the strength of the correlation between an item and the latent trait under investigation; and *H*_s_ (scale), measuring the quality of total scale, a weighted mean of item coefficients, an index for the precision of ordering person. According to Ligtvoet et al. (2010), the cut-off points are: unscalable (*H*_i_, *H*_s_ < 0.3), poor scalability (0.3 < *H*_i_, *H*_s_ < 0.4), moderate scalability (0.4 < *H*_i_, *H*_s_ < 0.5), and strongly scalability (*H*_i_, *H*_s_ > 0.5).

Mokken scale analysis was conducted to explore whether there were hierarchical properties in users’ perceptions and of the AVUPS. First, all 28 AVUPS items were entered into an AISP and any item with a H_i_ scalability coefficient below 0.3 was removed. Then scale portioning was carried out to explore the dimensions of users’ perceptions through increasing *c*—where the lower bound *c* defines the minimum value of coefficients *H*_i_ in the Mokken scale by 0.05 increments. Then, the MHM assumptions were investigated at sub-scale level (i.e., factor constructs from the EFA) and at a scale level. The second assumption of local independence was checked using a conditional association procedure (Straat, 2012) and locally dependent items were removed. The third assumption of monotonicity was checked using item-rest regression (Sijtsma and Molenaar, 2002; Mokken, 2011). Items were removed if they violated monotonicity with a *crit* statistic >40 (Molenaar and Sijtsma, 2000). Lastly, test–retest reliability of AVUPS was assessed using intra-class correlation (ICC), Bland-Altman plot method, and paired sample correlation (Sackett et al., 1985).

## Results

A normality check was performed for each item by computing the univariate skewness (>3) and kurtosis (>10) (Kline, 2010). The skew indexes ranged from −2.05 to 0.86, the kurtosis indexes range from −1.26 to 5.80. The Kaiser-Meyer-Olkin (KMO) measure of sampling adequacy suggests that data seems appropriate for factor analysis, KMO = 0.94. Bartlett’s test of sphericity suggested that there is sufficient significant correlation in the data for an EFA, χ^2^ (378) = 5739.23, *p* < 0.001.

### Measurement Model

#### Exploratory Factor Analysis

An EFA was performed on all 28 AVUPS items to compare the factor structure of the empirical data against the conceptual model (11 Factors; see Figure 1). The EFA was built using Principal Axis Factoring (PAF) method and oblimin rotation. There were signs of low-loading (cutoff: <0.4) and the factor structure did not match the conceptual model. Thus, a parallel analysis was performed to determine the number of factors to keep in the EFA. Using eigenvalues (cutoff: <1), the parallel analysis suggested four factors for the AVUPS. A follow-up EFA (Table 1), displayed signs of low-loading items, resulting in two items (Items 18 and 23) being excluded from the subscales (i.e., factors). The four-factor structure with 26 items, explaining 57.35% of the variance, conceptually represented *IU* (13 items), *perceived barriers* (PB) (7 items), *well-being* (4 items), and *experience with technology* (2 items). The factor labels were determined by assessing item content, commonalities, and Loevinger’s coefficient of homogeneity (Fabrigar et al., 1999).

**TABLE 1:** Principal axis factoring with oblimin rotation.

		Factor			
Item	M (SD)	Intention to use	Perceived barriers	Well-being	Experience with technology
AVUPS 1	86.1 (16.3)				0.59
AVUPS 2	73.5 (24.3)				0.48
AVUPS 3^R^	70.4 (28.2)		0.60		
AVUPS 4	78.2 (27.0)	0.64			
AVUPS 5^R^	63.0 (32.0)		0.55		
AVUPS 6	65.2 (27.0)	0.62			
AVUPS 7	56.0 (31.9)	0.59			
AVUPS 8	68.0 (29.3)	0.73			
AVUPS 9	83.0 (20.7)	0.51			
AVUPS 10	65.0 (28.8)			0.78	
AVUPS 11	58.4 (32.7)			0.83	
AVUPS 12	69.7 (27.9)			0.50	
AVUPS 13	78.4 (21.8)	0.42			
AVUPS 14^R^	70.2 (28.8)		0.80		
AVUPS 15	66.3 (30.7)	0.74			
AVUPS 16^R^	68.8 (32.2)		0.46		
AVUPS 17^R^	53.4 (31.6)	0.66			
AVUPS 18^R^	29.6 (29.1)				
AVUPS 19^R^	56.4 (30.8)		0.46		
AVUPS 20	56.7 (32.8)	0.82			
AVUPS 21	75.2 (29.0)	0.77			
AVUPS 22	76.6 (27.7)	0.61			
AVUPS 23	61.2 (23.6)				
AVUPS 24	62.3 (25.1)			0.58	
AVUPS 25	69.8 (25.7)	0.62			
AVUPS 26^R^	71.6 (29.3)		0.60		
AVUPS 27	67.3 (26.3)	0.68			
AVUPS 28^R^	61.5 (32.7)		0.54		
Cronbach’s α		α = 0.94	α = 0.84	α = 0.87	α = 0.64
CR		0.93	0.86	0.86	0.76

*AVUPS, Automated Vehicle User Perception Survey; CR, composite reliability; ^R^, Item was reverse-coded.*

#### Internal Consistency of the AVUPS

Cronbach’s alpha (cutffoff: >0.8; Cronbach, 1951) and composite reliability (cutoff: >0.7; Hair et al., 1998) were used to assess the internal consistency of the items and each of its factors. Overall, the internal consistency of this scale was excellent (Cronbach’s α = 0.95) with factors ranging from moderate to excellent (range α = 0.64 – 0.94; Table 1). The overall Cronbach’s α would not be affected by removing any individual items from the scale, as new α’s maintained an α of 0.94 with the deletion of any individual item. Similarly, as shown in Table 1, the composite reliability measures (i.e., construct reliability) ranged from 0.76 to 0.93.

#### Mokken Scaling Analysis for Subscales

Mokken scaling analysis was performed on subscales (i.e., factors), with four or more items (Stochl et al., 2012), identified during the EFA (see Table 2). All 13 items in the first subscale, *IU*, had inter-item scalability coefficients (*H*_i_ range = 0.40–0.66) greater than 0.3 and thus no items were removed. There were no signs of local independence or monotonicity violations and the subscale was strong (*H*_s_ = 0.55) and reliable (ρ = 0.93). For the second subscale, *PB*, six of the seven items had adequate scalability coefficients (*H*_i_ range = 0.31–0.55), resulting in the removal of Item 3 (*H*_i_ < 0.3). There were no signs of local independence or monotonicity violations and the subscale was moderate (*H*_s_ = 0.47) and reliable (ρ = 0.84). All four items for the subscale, *well-being*, had adequate scalability coefficients (*H*_i_ range = 0.58–0.69) and displayed no violations of local independence or monotonicity. The subscale was strong (*H*_s_ = 0.66) and reliable (ρ = 0.88). The fourth factor, *experience with technology*, contained 2 items which was insufficient for MSA.

**TABLE 2:** Mokken scaling with items.

Subscales				AVUPS		
Items ordered by factors	Step 1 (H_i_)	Step 2	Subscale	Step 1 (H_i_)	Step 2	Scale
**Intention to Use**						
AVUPS 4	0.64		IU	0.63		AVUPS
AVUPS 6	0.61		IU	0.58		AVUPS
AVUPS 7	0.40		IU	0.35		AVUPS
AVUPS 8	0.54		IU	0.50		AVUPS
AVUPS 9	0.46		IU	0.42		AVUPS
AVUPS 13	0.53		IU	0.53		AVUPS
AVUPS 15	0.57		IU	0.54		AVUPS
AVUPS 17	0.50		IU	0.47		AVUPS
AVUPS 20	0.55		IU	0.51		AVUPS
AVUPS 21	0.66		IU	0.64		AVUPS
AVUPS 22	0.59		IU	0.57		AVUPS
AVUPS 25	0.60		IU	0.57		AVUPS
AVUPS 27	0.63		IU	0.60		AVUPS
**Perceived barriers**						
AVUPS 3	<0.3			<0.3		
AVUPS 5	0.52		PB	0.50		AVUPS
AVUPS 14	0.42		PB	<0.3		
AVUPS 16	0.52		PB	0.52		AVUPS
AVUPS 19	0.31		PB	<0.3		
AVUPS 26	0.50		PB	0.40	Violated	
AVUPS 28	0.55		PB	0.55		AVUPS
**Well-being**						
AVUPS 10	0.67		W	0.44		AVUPS
AVUPS 11	0.69		W	0.38	Violated	
AVUPS 12	0.69		W	0.59		AVUPS
AVUPS 24	0.58		W	0.42		AVUPS
**Experience with Technology**						
AVUPS 1				<0.3		
AVUPS 2				0.31	Violated	
**Did Not Scale**						
AVUPS 18				0.32		AVUPS
AVUPS 23				<0.3		

*IU, Intention to use; PB, perceived barriers; W, Well-being. Step 1 required H_i_ = 0.3. Step 2 explored monotonicity and local independence assumptions.*

#### Mokken Scaling Analysis

Mokken scale analysis was performed on all 28 items to determine if the scale is unidimensional (see Table 2). Based on the condition that inter-item scalability coefficients (*H*_i_ range = 0.21–0.64) should be greater than 0.3, 6 items were removed (Items 1, 2, 3, 14, 19, and 23) and 22 items remained. Then exploration was carried out on all 22 items. There was no violation of local independence for any of the 28 items. Monotonicity was violated and items were iteratively removed to meet the monotonicity assumptions. This resulted in the removal of 2 items (Items 11 and 26) from the scale. To explore the dimensions of the 20 items, lower bound c started from 0.05 and increased to 0.75 in 0.05 increments. From 0.05 to 0.30, all items formed a single scale after which an item was selected for removal at *c* = 0.35. A second scale did not emerge until *c* = 0.50. So the final solution to the Mokken scaling was set at *c* = 0.30. The 20-item AVUPS was strong (*H*_s_ = 0.51) and reliable (ρ = 0.95).

#### Test–Retest Reliability

**FIGURE 2:**
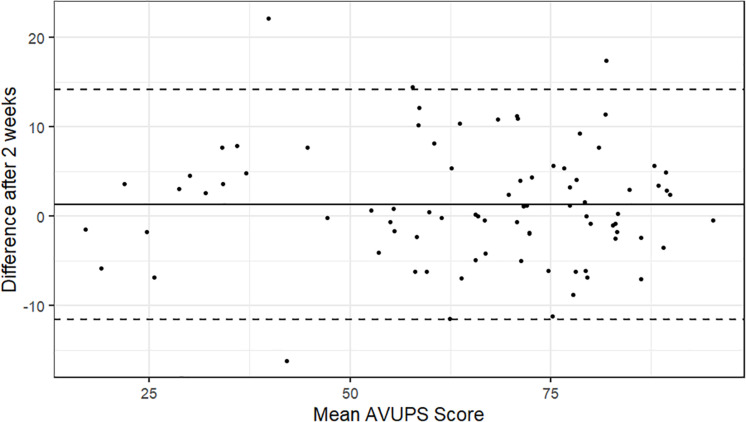
Bland-Altman plot: Intraindividual differences (*n* = 84) between mean AVUPS scores for test–retest, plotted against the average of the two scores. The central line represents the mean difference and the top and bottom lines display the 95% confidence interval.

A sample of 84 MTurk Workers was used to estimate the test–retest reliability of the AVUPS. Participants completed the AVUPS again, 2 weeks after the first AVUPS. The Bland-Altman plot method was used to visually inspect the test–retest reliability after 2 weeks (see Figure 2). As displayed in the plot, 4 (4.8%) of the 84 within-subject test–retest difference scores were outside of the 95% CI [−11.71, 14.03]. Spearman’s rho (ρ) and intraclass correlation coefficients (ICC_2_,_1_) were computed to assess the test–retest reliability at the subscale level. A perfect Spearman correlation of −1 or +1 occurs when the variables are a perfect monotone function of one another. ICC reliability values can range from 0 to 1 and can be interpreted as poor (<0.4), fair (0.4–0.6), good (0.6–0.75), and excellent (>0.75; Fleiss et al., 2013). The total AVUPS scores for test and retest reliability in these 84 participants were significantly and strongly correlated with excellent reliability (ρ = 0.76, *p* < 0.001, ICC = 0.95). The separate Mokken scale (i.e., factors) scores for test–retest were also significantly and strongly correlated with excellent reliability: *IU* (ρ = 0.80, *p* < 0.001, ICC = 0.93), *PB* (ρ = 0.73, *p* < 0.001, ICC = 0.87), and *well-being* (ρ = 0.72, *p* < 0.001, ICC = 0.84). The test–retest reliability for e*xperience with technology* was not assessed as the subscale was not validated using MSA. All individual items for the test and retest reliability correlated significantly, with paired sample correlations ranging from 0.56 to 0.89.

## Discussion

The purpose of this study was to report on the construct validity and reliability—specifically, scale validation, factor structure, and test–retest reliability of a new survey to assess adults’ perception of acceptance to AVs. The survey in this study was developed to quantify users’ perception of acceptance to fully AVs (SAE Levels 4,5). The scale was previously constructed to align with the conceptual model, which was empirically developed from seven acceptance models (Figure 1). Results from both the EFA and MSA suggested that the factor structure of the AVUPS did not align with the conceptual model. Interestingly, results from the EFA and MSA generated different factor structures. The EFA resulted in a four-factor structure with 26 items whereas the MSA was unidimensional and included 20 items. However, using the results from the EFA to inform MSA, resulted in the validation of three Mokken subscales (*IU*, *PB*, and *well-being)* and one whole scale (AVUPS). The 20-item AVUPS (i.e., whole scale) is a strong Mokken scale, which met the criteria for a MHM and displayed excellent internal consistency and excellent 2 weeks test–retest reliability.

The AVUPS and three Mokken subscales of AV acceptance has been identified using EFA and MSA in a moderate size sample data set of adults living in the United States. Mokken scale analysis provided comprehensive output about the scalability of items, validation of subscales, and unidimensionality of the whole scale. The EFA was useful in informing the MSA of potential subscales. Interestingly, these subscales did not emerge when entering all AVUPS items together into the MSA. Similar to validation by Chen et al. (2016), the approach of using EFA and MSA should be considered during scale development. Using the factor constructs from the EFA resulted in three subscales that were reliable and moderate to strong Mokken scales. These subscales will be important when assessing an individuals’ *IU*, *PB*, and *well-being.* The *experience with technology* subscale was not tested and the items were removed from the AVUPS due to inadequate scalability coefficients (*H*_i_). Conceptually, the two items are related to the users’ prior experience with technology and does not align with the AVUPS or subscales.

During initial scale development (Mason et al., 2020), nine AVUPS items were negatively worded and EFA results indicated one item did not scale, one item loaded on *IU* (Factor 1), and seven items loaded on *barriers* (Factor 2). The items were developed to avoid response biases associated with multi-item scales that are worded in a single direction (i.e., acquiescence) as suggested by several psychometricians (Churchill, 1979; Baumgartner and Steenkamp, 2001). However, reverse-coded items may produce artifactual response factors consisting exclusively of negatively worded items or reduce reliability by interfering with inter-item correlation (Harvey et al., 1985; Wong et al., 2003; Krosnick and Presser, 2010). Out of the nine negatively worded items, six items were included in the AVUPS Mokken scale. The second subscale, *barriers*, included six items that were conceptually developed to represent trust, perceived ease of use, IU, driving self-efficacy, and safety. These six items were negatively worded and reverse-coded/scored. Two out of the six items were self-generated whereas the other four items were modified from previous technology acceptance surveys (Gold et al., 2015; Nees, 2016; Cho et al., 2017). In this study, consequences from negatively worded items were not evident as not all reverse-coded items loaded onto one scale. Furthermore, the subscales and AVUPS had excellent internal consistency and 2 weeks test–retest reliability.

The subscale from the first factor, *IU*, consists of the largest number of items (13 items) with items developed to represent concepts such as trust, perceived usefulness, IU, perceived ease of use, cost, authority, and safety. Behavioral IU AVs can be predicted by attitudes and perceptions (Payre et al., 2014). Previous models have suggested that perceived usefulness and perceived ease of use have a large impact on IU (Davis, 1989; Madigan et al., 2017; May et al., 2017). This aligns with our findings with items loading into one subscale, *IU.* This Mokken subscale or any combination of subscales may be used to reduce respondent burden and provide support for research questions related to their respective construct. The third Mokken subscale, *well-being*, includes 4 items that can quantify adults’ perceptions of AVs and how this technology may influence their ability to stay active, participate in their community, and enhance their quality of life. Advanced driver assistance systems (ADAS) and AVs may increase drivers’ safety and mobility in their community (Reimer, 2014). However, these systems may be too complex (Yang and Coughlin, 2014), require hands-on training to promote the safe use of this emerging technology (Classen et al., 2019), or be perceived as only useful for drivers with poor or declining skills (Lefeuvre et al., 2008). Utilizing these scales to assess *a priori* perceptions and post-exposure perceptions may help to quantify users’ perceptions and lead to promoting the acceptance and adoption of AVs.

### Limitations

This survey lays an important foundation in assessing perceptions of *use or IU AVs*—which is not a guarantee for the actual acceptance and adoption of the AV. As such, developmental and empirical investigations are deemed necessary to provide substantive evidence for measuring and quantifying actual acceptance and adoption practices versus perceptions thereof. Future projects may consider measuring the individuals’ familiarity with AVs as this will likely influence their perceptions, understanding, and acceptance of AVs. Although not performed in this study, a non-essential but desirable feature of MSA is invariant item ordering (IIO; i.e., double monotonicity) whereby the order of items along the latent trait is the same for all respondents at all levels of the latent trait (Aleo et al., 2019). Once familiarity with AVs is assessed in a sample size of =500 respondents (Watson et al., 2018), the more restrictive MSA model, double monotonicity model, should be tested. Sample size is also potential limitation of this study as the minimum sample size requirement is a matter of debate in the field of MSA. As suggested by Watson et al. (2018), the current study met the minimal requirement of 250 respondents for AISP algorithms. Lastly, there are numerous factors that need to be considered when assessing perceptions of AVs such as socioeconomic status, technology literacy, environmental factors and culture, area of residence, shared vs private AVs, and the complex challenge that driving or community mobility is strongly context- and situation-dependent.

### Strengths

The authors utilized a multi-pronged approach to examine, quantify, and refine the psychometrics of the AVUPS. Both classical test theory and item response theory were used to enhance the current instrument. Multivariate statistical techniques such as Cronbach’s alpha and EFA fall under the classical test theory umbrella and were used to measure common variance between the variables. Next, a non-parametric item response theory, MSA, was used to compliment the EFA. This method primarily analyses the behavior of individual items, and based on their properties, investigates how they relate to other items. Item response theory (i.e., MSA) was used to establish a relationship between the score of an item and the score on the latent trait (Aleo et al., 2019). In the refined form, the AVUPS demonstrates potential for wide scale use to elicit the perceptions of adults pertaining to their *IU*, *PB*, and *well-being* related to AVs.

Next steps indicate advances in research, such as establishing the criterion validity of the AVUPS. In particular, if actual acceptance and adoption practices can be measured, we may regress these outcomes to the current variables to ensure that each item and each sub-scale are adequately contributing to accurately measuring such practices, among those adults who are using AVs. Our findings also indicate implications for policy. Specifically, city managers and transportation planners may benefit from using this survey to estimate users’ perceptions prior to actual deployment of AVs in the community, to better understand the citizens’ *IU*, *PB*, and *well-being* pertaining to AVs. State departments of transportation, especially those interested in life-long mobility, may benefit from using this survey to inform their long-range transportation planning practices. Lastly, automotive industry involved in development of AVs may benefit by tailoring their designs to the perceptions of future users.

## Conclusion

The approach adopted in this study and the initial survey development (Mason et al., 2020) ensured that the survey instrument design included items that were relevant, concise, and clear. Specifically, the conceptual model guided item generation from the extant literature, followed by an assessment to determine face validity, and two rounds of reviews from subject-matter experts to establish content validity. The validation of the AVUPS and three separate Mokken subscales, the whole scale can be utilized or any combination of separate subscales to quantify users’ perceptions of AVs. Future research may be performed to establish criterion validity, replicate the dimensionality, and to determine whether similar items demonstrate invariant item ordering. Currently, the survey may be utilized to assess road users’ acceptance of AVs and potentially predict their IU this innovative technology. Furthermore, this instrument also holds potential for informing city managers and transportation planners of the public’s opinion on fully AVs.

## Data Availability Statement

The raw data supporting the conclusions of this article will be made available by the authors, without undue reservation.

## Ethics Statement

The studies involving human participants were reviewed and approved by University of Florida Institutional Review Board. The patients/participants provided their written informed consent to participate in this study.

## Author Contributions

JM and SC: study conceptualization and design. JM, SC, and VS: survey development. JM and JW: data collection. JM: analysis and interpretation of results. JM, SC, VS, and JW: draft manuscript preparation. All authors reviewed the results and approved the final version of the manuscript.

## Conflict of Interest

The authors declare that the research was conducted in the absence of any commercial or financial relationships that could be construed as a potential conflict of interest.
