# Supplementary material for: Construct Validity and Test–Retest Reliability of the Automated Vehicle User Perception Survey
Source: Front Psychol. 2021 Jan 25;12:626791. doi: 10.3389/fpsyg.2021.626791 (PMC7868437; doi:10.3389/fpsyg.2021.626791)
Supplement: Supplementary file 1 [file Table_1.docx]

| Item # | Item |
| --- | --- |
| 1 | I use technology to make tasks easier for me |
| 2 | I use technology in my vehicle to make tasks easier for me |
| 3 | I have had bad experiences when I try to use new technology instead of doing things “the old-fashioned way” |
| 4 | I am open to the idea of using automated vehicles |
| 5 | I am suspicious of automated vehicles |
| 6 | I believe I can trust automated vehicles |
| 7 | I will engage in other tasks while riding in an automated vehicle |
| 8 | I believe automated vehicles will reduce traffic congestion |
| 9 | I believe automated vehicles will assist with parking |
| 10 | I believe automated vehicles will allow me to stay active |
| 11 | Automated vehicles will allow me to stay involved in my community |
| 12 | Automated vehicles will enhance my quality of life/well-being |
| 13 | I expect that automated vehicles will be easy to use |
| 14 | It will require a lot of effort to figure out how to use an automated vehicle |
| 15 | I would use an automated vehicle on a daily basis |
| 16 | I would rarely use an automated vehicle |
| 17 | Even if I had access to an automated vehicle, I would still want to drive myself |
| 18 | It will be important to have the option to drive myself by turning off the automated system |
| 19 | My driving abilities will decline due to relying on an automated vehicle |
| 20 | I will be willing to pay more for an automated vehicle compared to what I would pay for a traditional car |
| 21 | If cost was not an issue, I would use an automated vehicle |
| 22 | I would use an automated vehicle if National Highway Traffic Safety Administration (NHTSA) deems them as being safe |
| 23 | Media portrays automated vehicles in a positive way |
| 24 | My family and friends will encourage/support me when I use an automated vehicle |
| 25 | When I’m riding in an automated vehicle, other road users will be safe |
| 26 | I believe that automated vehicles will increase the number of crashes |
| 27 | I feel safe riding in an automated vehicle |
| 28 | I feel hesitant about using an automated vehicle |
