# Supplementary material for: Construct Validity and Test–Retest Reliability of the Automated Vehicle User Perception Survey
Source: Front Psychol. 2021 Jan 25;12:626791. doi: 10.3389/fpsyg.2021.626791 (PMC7868437; doi:10.3389/fpsyg.2021.626791)
Supplement: Supplementary file 2 [file Table_2.docx]

**Autonomous Vehicle User Perception Survey**

Definition: *An automated vehicle (i.e., self-driving vehicle, driverless car, self-driving shuttle) is a vehicle that is capable of sensing its environment and navigating without human input. Full-time automation of all driving tasks on any road, under any conditions, and does not require a driver nor a steering wheel.*

Directions: *Please place a vertical dash ( / ) on the scale to display the degree to which you agree or disagree with the statement.*

1. I am open to the idea of using autonomous vehicles *(AVUPS 4)*

Disagree Agree

1. I am suspicious of autonomous vehicles *(AVUPS 5)*

Disagree Agree

1. I believe I can trust autonomous vehicles *(AVUPS 6)*

Disagree Agree

1. I will engage in other tasks while riding in an autonomous vehicle *(AVUPS 7)*

Disagree Agree

1. I believe autonomous vehicles will reduce traffic congestion *(AVUPS 8)*

Disagree Agree

1. I believe autonomous vehicles will assist with parking *(AVUPS 9)*

Disagree Agree

1. I believe autonomous vehicles will allow me to stay active *(AVUPS 10)*

Disagree Agree

1. Autonomous vehicles will allow me to stay involved in my community *(AVUPS 11)*

Disagree Agree

1. Autonomous vehicles will enhance my quality of life/well-being *(AVUPS 12)*

Disagree Agree

1. I expect that autonomous vehicles will be easy to use *(AVUPS 13)*

Disagree Agree

1. It will require a lot of effort to figure out how to use an autonomous vehicle *(AVUPS 14)*

Disagree Agree

1. I would use an autonomous vehicle on a daily basis *(AVUPS 15)*

Disagree Agree

1. I would rarely use an autonomous vehicle *(AVUPS 16)*

Disagree Agree

1. Even if I had access to an autonomous vehicle, I would still want to drive myself *(AVUPS 17)*

Disagree Agree

1. It will be important for me to have the option to drive myself by turning off the autonomous system *(AVUPS 18)*

Disagree Agree

1. My driving abilities will decline due to relying on an autonomous vehicle *(AVUPS 19)*

Disagree Agree

1. I will be willing to pay more for an autonomous vehicle compared to what I would pay for a traditional car *(AVUPS 20)*

Disagree Agree

1. If cost was not an issue, I would use an autonomous vehicle *(AVUPS 21)*

Disagree Agree

1. I would use an autonomous vehicle if National Highway Traffic Safety Administration (NHTSA) deems them as being safe *(AVUPS 22)*

Disagree Agree

1. My family and friends will encourage/support me when I use an autonomous vehicle *(AVUPS 24)*

Disagree Agree

1. When I’m riding in an autonomous vehicle, other road users will be safe *(AVUPS 25)*

Disagree Agree

1. I believe that autonomous vehicles will increase the number of crashes *(AVUPS 26)*

Disagree Agree

1. I feel safe riding in an autonomous vehicle *(AVUPS 27)*

Disagree Agree

1. I feel hesitant about using an autonomous vehicle *(AVUPS 28)*

Disagree Agree

1. Describe influences that may promote your willingness to use autonomous vehicles *(AVUPS 29)*
2. Describe influences that may deter you from using autonomous vehicles *(AVUPS 30)*
3. Describe potential benefits of autonomous vehicles *(AVUPS 31)*
4. Describe potential disadvantages of autonomous vehicles *(AVUPS 32)*
